# Cationic Polymer Brushes Functionalized with Carbon Dots and Boronic Acids for Bacterial Detection and Inactivation

**DOI:** 10.1021/acsomega.5c01507

**Published:** 2025-04-02

**Authors:** Qicheng Zhang, Si Chen, Xiaoting Xue, Solmaz Hajizadeh, Tomohiko Yamazaki, Lei Ye

**Affiliations:** † Division of Pure and Applied Biochemistry, Department of Chemistry, Lund University, Lund 22100, Sweden; ‡ Polymer & Materials Chemistry, Department of Chemistry, Lund University, Lund 221 00, Sweden; § Research Center for Macromolecules and Biomaterials, National Institute for Materials Science (NIMS), Tsukuba 305-0047, Japan

## Abstract

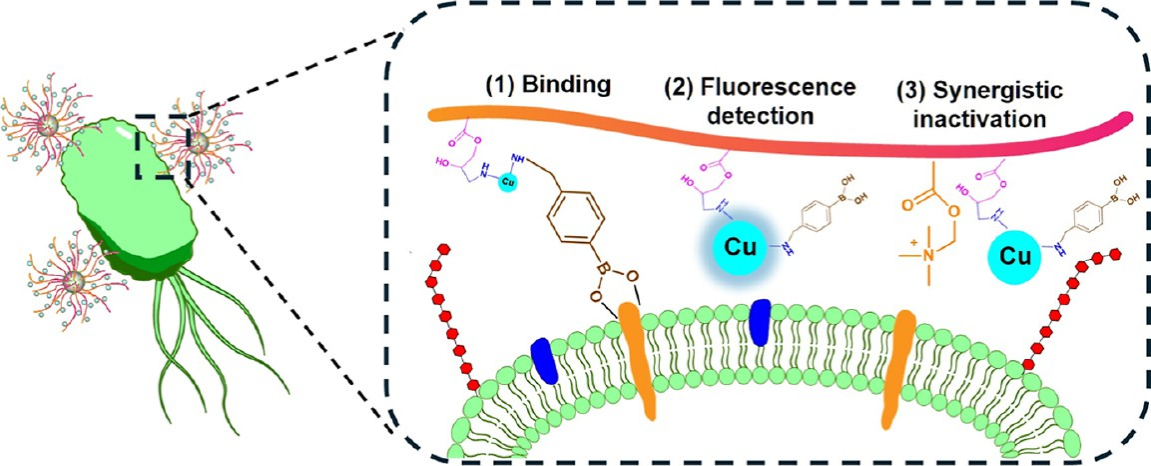

Drug-resistant bacterial infections are among the most severe physiological challenges facing human health. Therefore, the detection and inactivation of pathogenic bacteria remains a crucial therapeutic goal in modern society. In this study, we design multifunctional nanocomposites aimed at bacterial binding, fluorescence labeling, and synergistic antibacterial treatment. These nanocomposites are prepared by introducing cationic polymers with quaternary ammonium compounds onto silica nanoparticles using surface-initiated atom transfer radical polymerization, followed by incorporation of copper-doped carbon dots and modification of boronic acid. The cationic polymer units and boronic acid end groups enhance the bacterial binding capacity and synergistic bactericidal effects in cooperation with the carbon dots. Due to the stable fluorescent properties of carbon dots, the nanocomposites can generate fluorescence signals around bacteria, enabling bacterial fluorescence imaging. Overall, this study demonstrates a multifunctional nanocomposite-assisted strategy for bacterial labeling, imaging, and deactivation, providing a novel approach for bacterial detection and synergistic treatment.

## 1 Introduction

Bacteria-induced infections are among the most severe medical problems, causing many serious diseases such as plague, sepsis, and infective endocarditis.^1,2^ The spread of such infections can also result in substantial economic losses in poultry production and food industry, increasingly attracting public attention worldwide.^3,4^ Naturally, the detection and deactivation of pathogenic bacteria are essential for responding to bacterial threats. However, this remains a significant challenge and a practical issue of great importance.

Antibiotic therapies have been widely used to combat microbial infections, but many pathogens have evolved drug resistance due to the overuse of these antibacterial agents.^5−7^ Therefore, new approaches to prevent the emergence of resistant bacteria, such as cationic compounds (or polymers) with positive charges, photothermal materials that release heat energy, and inhibitors that disrupt microbial quorum sensing, have been widely explored.^8−10^ There are several alternative treatments that destroy pathogens by generating reactive oxygen species under different trigger conditions, such as lasers (photodynamic therapy) and certain transition metal elements (chemodynamic therapy).^11,12^ Additionally, antimicrobial peptides, which naturally form polypeptide sequences composed of cationic and hydrophobic amino acids with direct antibacterial activity, are also powerful strategies to address the impending crisis of antimicrobial resistance.^13^

Another critical issue is the selection of analytical technology for the detection of bacteria. Compared with the time-consuming and labor-intensive bacterial colony-forming unit counting method, fluorescence-based techniques offer low cost, fast response, and easy accessibility.^14,15^ Moreover, the detection process of bacteria inevitably involves bacterial binding. Boronate affinity techniques have emerged as a powerful tool in bioconjugation, molecular identification, and chromatographic separation due to the formation of boronate ester complexes between boronic acid and the *cis*-diol structures of polysaccharides and oligosaccharides on the surface of pathogens, which can help us avoid the use of expensive antibodies.^16−19^ Therefore, designing a method that can noninvasively and efficiently bind, detect, and inactivate bacteria using fluorescent detection and boronate affinity is of great significance.^20−22^

To achieve this goal, the strengths of various bacteria-related approaches should be integrated into a single platform. Polymers are ideal nanoarchitectures for functionalizing and fabricating multipurpose materials. Polymers prepared using controlled radical polymerization (CRP) methods can be efficiently designed, endowing them with well-defined block sequences and desired molecular weights.^23−26^ Among various CRP methods, atom transfer radical polymerization (ATRP) is widely used to create new copolymer materials with diverse functionalities and precisely controlled molecular weights.^27,28^ When an initiator is prefixed onto a support, surface-initiated atom transfer radical polymerization (SI-ATRP) can be conducted to conveniently graft various types of functionalized polymer brushes onto solid substrates, such as silica nanoparticles, magnetic beads, metallic surfaces, and engineered scaffolds.^29−32^ The solid substrates facilitate straightforward purification of the polymer products through simple filtration or sedimentation. Typically, these polymers consist of two distinct functional parts containing different chemical groups and chain segments with different functions. For example, cationic monomers that carry quaternary ammonium compounds (QACs), such as [2-(methacryloyloxy)-ethyl]trimethylammonium chloride (METAC), have emerged as potential antibacterial building blocks to prepare the polymer brushes.^33,34^ The cationic polymers inactivate bacteria through strong interactions with the cytoplasmic membrane and irreversible destruction of the bacterial membrane integrity. Glycidyl methacrylate (GMA) is an excellent tunable monomer because its epoxy group can undergo a ring-opening reaction to enable conjugation of additional small molecules and even biomacromolecules onto polymer chains.^35,36^ Polymer coatings composed of QACs monomers and GMA can be chemically modified to enhance antibacterial activity and add new functionalities.

As a zero-dimensional carbon material with abundant surface functional groups and excellent luminescence features, carbon quantum dots (CDs) have shown great potential in biolabeling and antibacterial fields.^37,38^ Generally, the inner core of CDs is composed of sp^2^ hybridized carbon, and the outer shell has organic functional groups. CDs can be doped with various heteroatoms and metal atoms to enhance the delocalization of electrons and the physicochemical properties of CDs.^39^ Compared to CDs doped with heteroatoms, metal-doped CDs exhibit higher surface charge and enhanced electron transfer, damaging bacteria through charge-induced physical destruction and reactive oxygen species-triggered oxidative stress.^40,41^ Simultaneously, the surface of CDs can be designed to have various functional groups by using different precursors, enabling CDs to be further modified with specific molecular recognition entities for versatile applications.^42,43^ Furthermore, CDs demonstrate an appealing and stable fluorescence nature originating from luminescent conjugated units composed of isolated sp^2^ carbon clusters. By adjusting structural differences, contributions from the carbon core and surface states, and the presence of final fluorophores, the photoluminescence of CDs can be effectively shifted to meet various bacterial imaging and detection needs.^44−46^

**Scheme 1:**
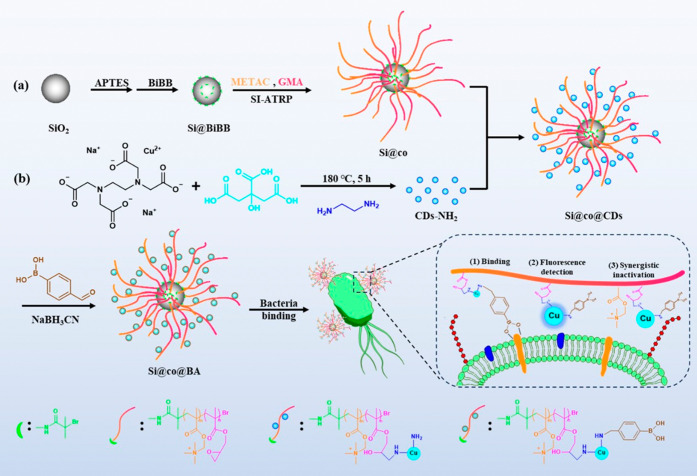
Schematic Illustration for the Synthesis of the Multifunctional Nanocomposite Si@co@BA and Application of the Material for Binding, Detection, and Inactivation of Bacteria

This work designed a new nanohybrid material to provide cellular binding, fluorescence labeling, and synergistic antibacterial activity by combining copolymer brushes grafted onto amino-functionalized silica with copper-doped CDs (Scheme 1). First, SI-ATRP was used to coat the poly(METAC-*co*-GMA) copolymer on silica, which endowed the nanocomposites with QACs-mediated antibacterial activity and reactive sites for introducing other functional materials. Subsequently, copper-doped CDs containing amino groups were linked to the copolymer chain through a ring-opening reaction of the epoxy groups (GMA) and amino groups (CDs), achieving a fluorescent capability and enhanced antibacterial activity. By immobilizing copolymers and CDs onto silica nanoparticles, the polymer chains and CDs can be easily separated for further use and a longer retention time. Finally, the remaining amino groups could react with aldehyde groups on 4-formylphenylboronic acid (FPBA) to add boronic acid for binding bacteria. The bacterial binding was facilitated by the multiple boronic acid ligands that form reversible boronate ester bonds with the polysaccharides and oligosaccharides in microbial cells. The antibacterial activity, copolymer properties, and physicochemical characteristics of the nanocomposites were investigated. Fluorescence microscopy, bacterial transmission electron microscopy (TEM), and flow cytometry were also used to demonstrate the labeling and imaging functions of the nanocomposites.

## 2 Experimental Section

### 2.1 Materials

Tetraethylorthosilicate (TEOS), ammonium hydroxide (25%), (3-aminopropyl)-triethoxysilane (APTES), triethylamine (TEA), 2-bromoisobutyryl bromide (BIBB), [2-(methacryloyloxy)ethyl]trimethylammonium chloride solution (METAC, 75%), glycidyl methacrylate (GMA), *N*,*N*,*N*′,*N*″,*N*″-pentamethyldiethylenetriamine (PMEDTA), copper(II)bromide (CuBr_2_), l-ascorbic acid (AscA), citric acid, ethylenediamine, 4-formylphenyl-boronic acid (FPBA), sodium cyanoborohydride (NaBH_3_CN), methanol, toluene, tetrahydrofuran (THF), and alizarin red S (ARS) were purchased from Sigma-Aldrich. Copper disodium ethylenediaminetetraacetate (Na_2_[Cu(EDTA)]) was purchased from TCI (Tokyo, Japan). Yeast extract and agar were purchased from Merck. Tryptone was purchased from Duchefa Biochemie. Sodium chloride (NaCl), disodium hydrogen phosphate (Na_2_HPO_4_), potassium dihydrogen phosphate (KH_2_PO_4_), and potassium chloride (KCl) were purchased from Fisher Scientific. Lysogeny broth (LB) media were prepared by dissolving yeast extract (5 g/L), tryptone (10 g/L), and NaCl (10 g/L) in water. Agar plates were prepared by adding extra agar (15 g/L) to the LB media.

### 2.2 Synthesis of Initiator-Modified Silica Nanoparticles (Si@BiBB)

Silica nanoparticles (SiO_2_) were prepared using a “one-step” Stöber reaction.^47^ Typically, 33 mL of water, 100 mL of methanol, and 22.4 mL of ammonium hydroxide (25%) were added into a 500 mL round-bottom flask and agitated at room temperature. Subsequently, a mixture of 130 mL of methanol containing 13.8 mL of TEOS was quickly added to the solution, which was then stirred for 8 h. The silica nanoparticles were separated by centrifugation at 10,000 rpm for 10 min and washed several times with water and methanol to remove unreacted ammonia and TEOS. The obtained nanoparticles were vacuum-dried at room temperature for further use.

To obtain amino-functionalized nanoparticles (SiO_2_@NH_2_), the silica nanoparticles (3.6 g) were dispersed in 120 mL of anhydrous toluene, followed by addition of 1.2 mL of APTES. The reaction mixture was vigorously stirred for 24 h at reflux temperature. The amino-functionalized nanoparticles were collected by centrifugation, washed several times with methanol and acetone, and treated as described above.

Si@BiBB was prepared using the following method: 500 mg of SiO_2_@NH_2_ and 0.8 mL of triethylamine were dispersed in 24 mL of THF, and the mixture was placed in an ice bath. Next, 1.15 g of BiBB was added dropwise to the suspension. The reaction mixture was warmed to ambient temperature and stirred overnight. The initiator-modified nanoparticles were isolated by centrifugation, washed several times with methanol and water, and dried in a vacuum desiccator overnight for further use.

### 2.3 Synthesis of Copolymer Brushes Grafted on Silica Nanoparticles (Si@co)

Si@BiBB nanoparticles (100 mg) were added to a 25 mL flask and dispersed in 4 mL of methanol by sonication. After addition of CuBr_2_ (8 mg), METAC (250 μL, 1 mmol), GMA (140 μL, 1 mmol), and PMDETA (7.4 μL) dissolved in 3 mL of water, the mixed solution was bubbled with nitrogen for 15 min to remove oxygen. Subsequently, the reaction solution was mixed with 1 mL of AscA (8 mg) to form the CuBr/CuBr_2_/PMDETA ATRP catalyst. The reaction mixture was bubbled with nitrogen gas for another 15 min, then sealed and heated to 60 °C for 24 h under a nitrogen atmosphere. The products were isolated and purified using the same procedures as described in Section 2.2.

### 2.4 Synthesis of Copper-Doped Carbon Dots

Typically, 1.05 g of citric acid, 500 mg of Na_2_[Cu(EDTA)], and 5 mmol of the amine precursor ethylenediamine were dissolved in 10 mL of water in a Teflon hydrothermal synthesis reactor. The autoclave was placed in a muffle furnace and heated to 200 °C for 5 h. After the reactor was cooled to ambient temperature, the suspension was centrifuged at 6000 rpm for 5 min and filtered with a 0.45 μm filter membrane to remove agglomerated particles. The filtrate was transferred into a dialysis bag (MWCO = 500 Da) and dialyzed against water for 24 h. CDs were obtained by freeze-drying the purified suspension and then stored in a vacuum desiccator.

### 2.5 Preparation of Polymer Brushes Modified with CDs (Si@co@CDs)

Briefly, 50 mg of Si@co and 25 mg of CDs were dispersed in 1 mL of methanol and water, respectively. Next, these two suspensions were mixed and heated to 60 °C for 24 h. The product was isolated and purified using the same procedures as those described in Section 2.2.

### 2.6 Preparation of Polymer Brushes Modified with CDs and Boronic Acids (Si@co@BA)

To introduce boronic acid groups onto Si@co@CDs particles, Si@co@CDs (30 mg), FPBA (10 mg), and NaBH_3_CN (5 mg) were dispersed in 2 mL of ethanol. The mixed dispersion was magnetically stirred at room temperature for 24 h, and the products were collected using the same procedures as described in Section 2.2.

### 2.7 Characterization

Transmission electron microscopy (TEM) characterization was carried out using a JEM-1400Plus microscope (JEOL, Japan). Scanning electron microscopy (SEM) was performed using a JSM-6700F instrument (JEOL, Japan). Fourier transform infrared (FT-IR) spectroscopy was conducted with a Nicolet iS5 instrument (ThermoFisher Scientific Inc., Waltham, USA). UV–vis spectroscopy was performed using either a Cary 60 UV–vis spectrophotometer (Agilent Technologies, USA) or a Varioska LUX multimode microplate reader (ThermoFisher Scientific Inc., Waltham, USA). Fluorescence spectroscopy was conducted using either a Cary Eclipse fluorescence spectrophotometer (Agilent Technologies, USA) or a Varioska LUX multimode microplate reader (excitation filter: 340–380 nm; dichromatic mirror: 400 nm; suppression filter: 435–485 nm). Dynamic light scattering (DLS) and zeta potential measurements were carried out by using a Zetasizer Nano ZS instrument (Malvern Instruments, UK). Nuclear magnetic resonance (NMR) spectroscopy measurements were performed with a Bruker DRX400 spectrometer at a proton frequency of 400.13 MHz. Thermal gravimetric analysis (TGA) was conducted using a TGA Q500 Thermogravimetric Analyzer in an air atmosphere. Fluorescent imaging was performed using a Nikon Eclipse Ci fluorescence microscope (Nikon, Japan). ICP–MS analysis was conducted using an Agilent 7850 ICP–MS.

## 3 Results and Discussion

### 3.1 Synthesis and Characterization of Nanocomposites

**Figure 1:**
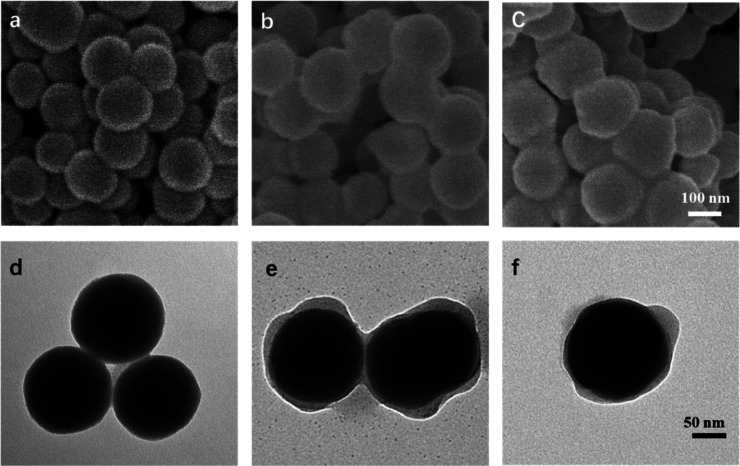
SEM and TEM images of (a,d) Si@BiBB, (b,e) Si@co, and (c,f) Si@co@BA. Scale bar in (a–c): 100 nm; (d–f): 50 nm.

The size and surface morphology changes at each step during the synthesis of multifunctional nanocomposites were investigated using SEM, TEM, and DLS. As shown in Figure 1a and d, the initiator-modified silica nanoparticles were spherical with a uniform diameter of around 141 nm, which corresponds to the hydrodynamic diameter data in Figure S1a (approximately 162 nm). After grafting METAC-GMA copolymer brushes onto the Si@BiBB surface, the Si@co nanocomposites exhibited a core–shell structure with a prominent copolymer shell (∼5 nm) visible in Figure 1e. The average diameter of Si@co was determined to be 150 nm (Figure 1b). However, the hydrodynamic diameter of Si@co appeared to be significantly larger (approximately 700 nm) than that of Si@BiBB, suggesting that the polymer brushes attached to the nanoparticles were hydrated and stretched in solution (Figure S1b). As shown in Figure S2, the morphology of Si@co@CDs showed no obvious difference compared to Si@co under an electron microscope due to the minuscule size of the CDs. However, the hydrodynamic diameter of Si@co@CDs decreased by approximately 150 nm after immobilization of the CDs, as the CDs reacted with the epoxy groups and made the nanocomposites became more hydrophilic and uniformly dispersed. Additionally, similar morphology and size can be observed when Si@co@BA was inspected compared with Si@co (Figures 1c,f and S1c).

**Figure 2:**
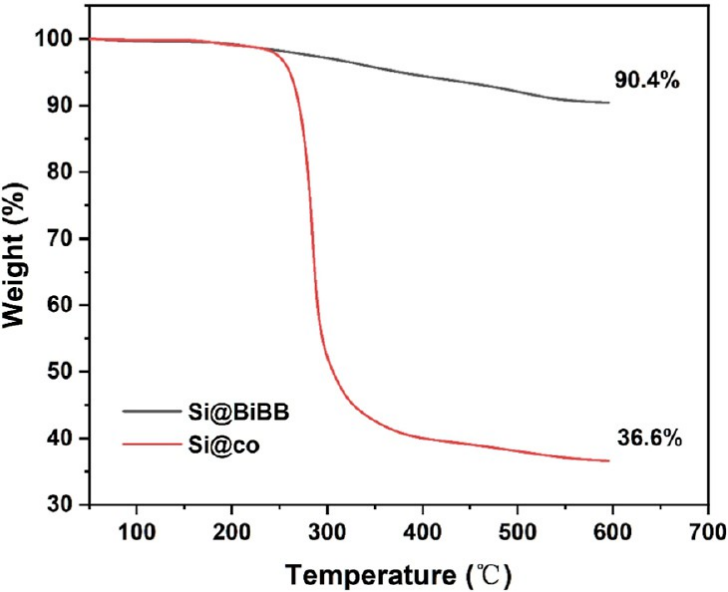
TGA analysis of Si@BiBB and Si@co.

The amount and chemical composition of the copolymer in the nanocomposite were analyzed by using TGA and ^1^H NMR. As shown in Figure 2, the TGA curve slightly declined below 250 °C due to the evaporation of residual organic solvent and water during the heating process. When the temperature increased to above 250 °C, an approximate 8.4% weight loss occurred in Si@BiBB, which can be attributed to the surface modification by APTES and BiBB molecules. Si@co exhibited a significant weight loss of around 63.4% as the temperature increased from 250 to 300 °C. This substantial weight loss is caused by the thermal decomposition of organic copolymers on the core–shell nanocomposites. Consequently, the amount of copolymer in Si@co was calculated to be 52.8% based on the difference in weight loss between Si@BiBB and Si@co. In additional TGA experiments, we found that the difference of weight loss between Si@BiBB and Si@NH_2_ at 600 °C was 1.1%, indicating that the amount of the initiator immobilized on the silica nanoparticles was around 0.073 mmol per gram of silica. If all the immobilized BiBB molecules contributed to initiate the polymerization, the average molecular weight of poly(METAC-*co*-GMA) grafted on the silica nanoparticles is calculated to be around 14,000 Da.

Moreover, the copolymer content was further explored using ^1^H NMR spectroscopy in DMSO-*d*_6_ (Figure S3). The methyl and methylene groups from GMA exhibited two peaks at 2.07 and 4.13 ppm (green and yellow dots), and the proton signal of terminal CH–CH_2_–O at 2.35 ppm overlapped with the DMSO-*d*_6_ peak at 2.49 ppm. For the METAC unit, the ^1^H NMR single peak of the methyl group and multiple peaks of the methylene group appeared at 1.24 and 5.77 ppm (red and blue dots), while the peak of the methyl groups from the quaternary ammonium group coincided with the water peak in DMSO-*d*_6_ at 3.3 ppm. Consequently, the final ratio of METAC to GMA in poly(METAC-*co*-GMA) was calculated to be approximately 1:1.6 based on the relative intensities of the ^1^H NMR peaks, indicating that fewer METAC monomers were incorporated into the polymer chains during the ATRP copolymerization.

**Figure 3:**
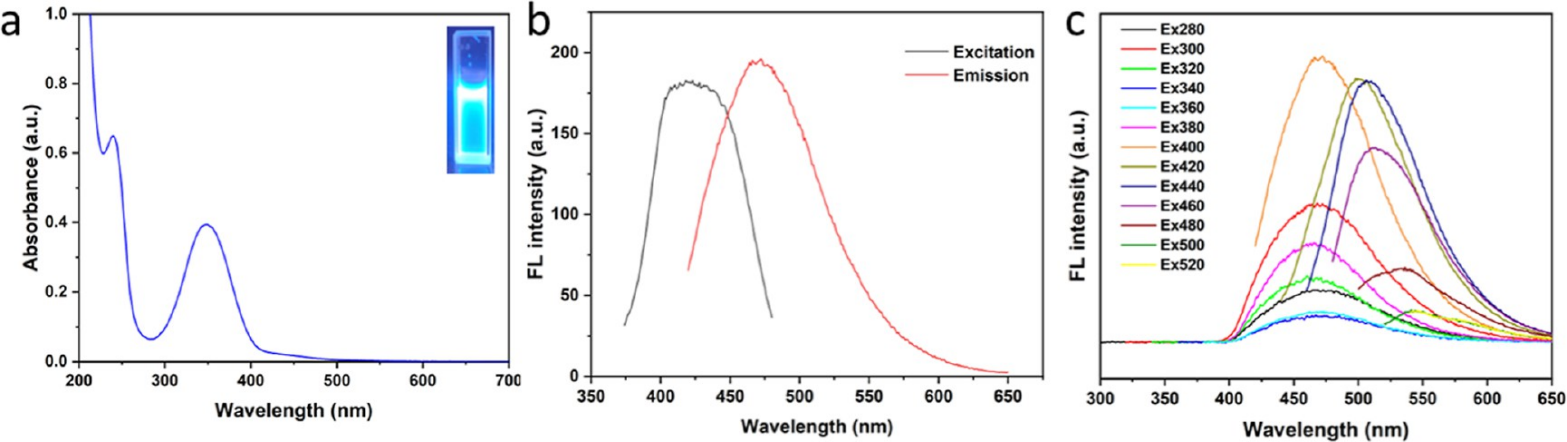
(a) UV–vis spectra of CDs. (b) Maximum fluorescence excitation and emission fluorescent spectra of CDs (excitation: 400 nm). (c) Excitation wavelength dependence of CDs.

The optical properties of the copper-doped CDs were examined by using UV–vis absorption and fluorescence spectroscopy. Figure 3a presents the steady-state absorption spectra of hybrid CDs. The as-prepared CDs displayed a shoulder peak in the high-energy range (240 nm) and a broad peak at 347 nm, which can be assigned to the π–π* transitions of sp^2^-hybridized carbon and n–π* transitions at the edge of the carbon lattice, respectively. In the wavelength region of 400–550 nm, a characteristic broad and low-strength absorption of CDs appeared originating from low-energy sub-band gaps caused by surface defects.

Photoluminescence (PL) spectra of CDs are shown in Figure 3b. The optimal excitation of the CDs is located at 420 nm, with an emission maximum wavelength at 470 nm, which endows the CDs with bright blue fluorescence in aqueous solution under 365 nm UV light. Additionally, by comparing the PL emission peaks under different excitation wavelengths ranging from 280 to 520 nm, the excitation-dependent emission properties of the CDs, which reflect important information related to different emission centers and transitions, were further investigated. As illustrated in Figure 3c, the PL peaks exhibited two separate regions: in the excitation range of 280–400 nm, the emission position remained at 470 nm, and PL intensities increased with the rise of the excitation wavelength, revealing the dominance of a single emissive transition. When the excitation moved above 400 nm, the main emission peaks shifted toward higher wavelengths (red-shifted) along with lowered intensities. These results demonstrate the excitation-wavelength-dependent feature and excellent PL properties of the CDs.

Based on the PL ability of CDs, the fluorescence spectra of Si@co@CDs and Si@co@BA were measured to investigate the CDs introduced into the nanocomposites (Figure S4a). Both Si@co@CDs and Si@co@BA exhibited strong fluorescence emission peaks at 470 nm under a maximum excitation wavelength, which agreed with the pure CDs, indicating that CDs had been successfully immobilized onto the polymer chains. ARS is commonly used to detect the presence of boronic acid due to the formation of fluorescent boronate ester products.^48^ To prove that the CDs had reacted with FPBA through the formation of a Schiff base between amino and aldehyde groups, the fluorescence spectra of Si@co@CDs-ARS and Si@co@BA-ARS complexes were tested by mixing the nanocomposites with ARS. As shown in Figure S4b, the color of the pinkish ARS solution was changed to orange by the Si@co@BA nanocomposite due to formation of the boronate ester, while the ARS color turned brownish after addition of Si@co@CDs. Moreover, Si@co@CDs-ARS displayed an obvious fluorescence emission centered at 540 nm due to the intrinsic fluorescence of CDs in the nanocomposites. After modification with boronic acid, the Si@co@BA particles were able to react with ARS to form a boronate ester complex, which showed a fluorescence emission peak red-shifted to 550 nm with an increased intensity under 469 nm excitation. Moreover, the UV absorption of ARS between 400 and 500 nm, the characteristic absorption region of the ARS-boronic acid complex, increased after Si@co@BA was added (Figure S4c). These results confirm the successful immobilization of CDs and boronic acid on the nanocomposites.

**Figure 4:**
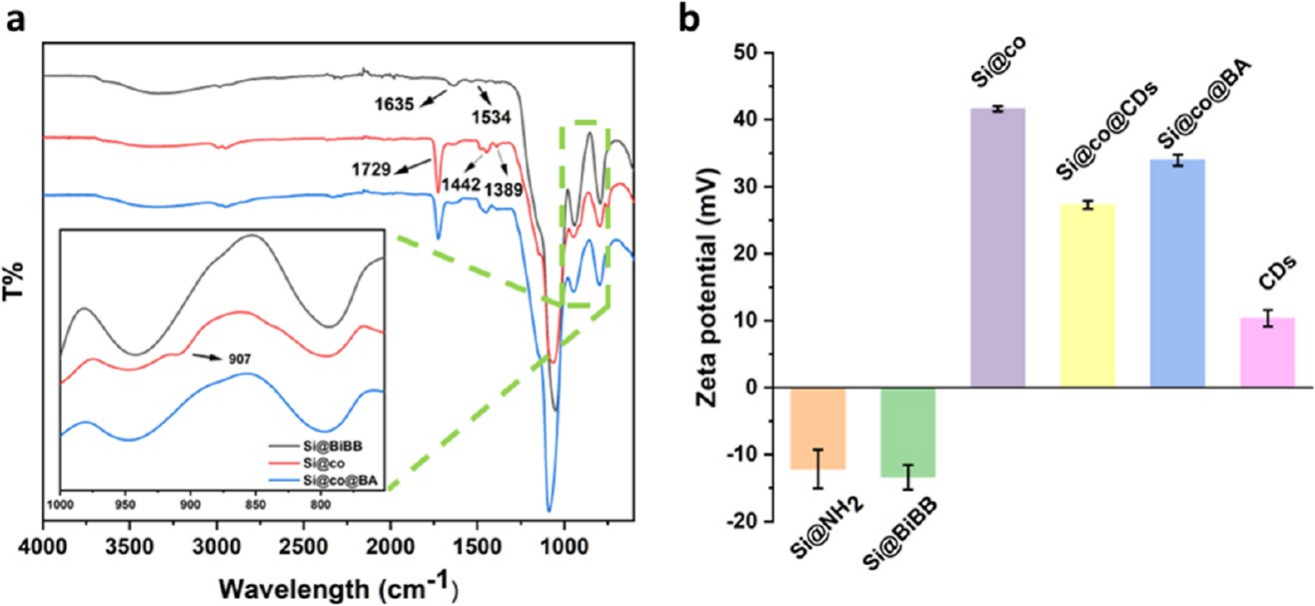
(a) FTIR spectra of Si@BiBB, Si@co, and Si@co@BA. (b) Zeta potential of Si@NH_2_, Si@BiBB, Si@co, Si@co@CDs, Si@co@BA, and CDs.

FT-IR spectroscopy was used to explore the chemical composition and functional group changes after each process step, as shown in Figures 4a and S5. For Si@NH_2_, absorption bands at 796, 949, and 1062 cm^–1^ corresponding to the Si–O–Si symmetrical stretching vibration, Si–OH bending vibration, and Si–O asymmetric stretching vibration, as well as a weak stretching vibration band of amino groups at 3348 cm^–1^, were observed. After the acylation reaction with the initiator, amide signals corresponding to the C=O stretching vibration at 1635 cm^–1^ and N–H stretching vibration at 1534 cm^–1^ appeared, indicating that the acylation reaction between BiBB and Si@NH_2_ occurred. After poly(METAC-*co*-GMA) was grafted onto the silica nanoparticles by SI-ATRP, a new band at 907 cm^–1^ assigned to the stretching vibration of epoxy groups from GMA and a band at 1442 cm^–1^ assigned to the bending vibration of the C–H bond from the quaternary ammonium groups were detected. Furthermore, a characteristic band corresponding to the stretching vibration of ester carbonyl groups at 1729 cm^–1^ from the two monomers also appeared in the IR spectra of Si@co. For the CDs, a strong N–H double peak signal around 3300 cm^–1^ can be seen, implying that the CDs possessed abundant amino groups able to react with the epoxy groups in the polymer brush.^49^ As a result, the band of epoxy groups at 907 cm^–1^ disappeared in Si@co@CDs after the introduction of CDs. The similar IR results between Si@co@CDs and Si@co@BA may be explained by the limited loading of boronic acid in the nanocomposite.

Zeta potential was measured to further confirm the successful modification during different stages, as shown in Figure 4b. Both Si@NH_2_ and Si@BiBB displayed a negative charge due to the abundant Si–OH groups on the silica nanoparticles. After GMA and METAC were polymerized onto the silica core, the surface charge increased sharply to 40 mV, caused by the positively charged QACs. Due to the slight positive charge of CDs, the Zeta potential of Si@co@CDs decreased slightly after the surface was occupied with part of the CDs, which was similar to that of Si@co@BA. Finally, the contents of copper and boron in the nanocomposites were analyzed by ICP–MS, which were determined to be 0.03% and 0.02%, respectively, confirming the presence of the metal and boronic acid in the nanocomposites.

### 3.2 Antibacterial Effect of Si@co@BA

**Figure 5:**
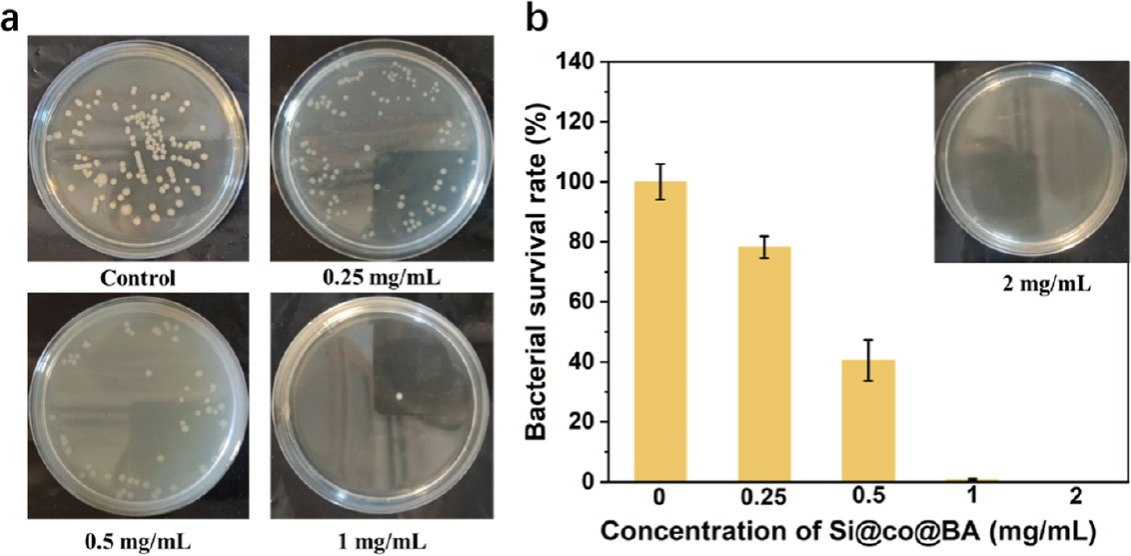
(a) Photographs and (b) corresponding cell survival rate of *Escherichia coli* treated with different concentrations of Si@co@BA.

Based on the antibacterial effect of the quaternary amines from the cationic copolymer and the copper dopant in the CDs, the synergistic antibacterial capability of the nanocomposites was investigated on *E. coli* using the plate counting method. As shown in Figure 5, the number of bacterial colonies decreased gradually as the concentration of Si@co@BA increased. When the concentration exceeded 1 mg/mL, Si@co@BA exhibited strong antibacterial effects against *E. coli* cells, as few bacterial colonies were observed on the corresponding agar plate. After calculation, the bactericidal rate was found to reach above 99% when the concentration of Si@co@BA was 1 mg/mL. The strong antibacterial activity of Si@co@BA can be attributed to the synergistic effect of the high content of METAC and copper dopant. Therefore, 1 mg/mL was selected as the concentration of the antibacterial material in subsequent experiments.

**Figure 6:**
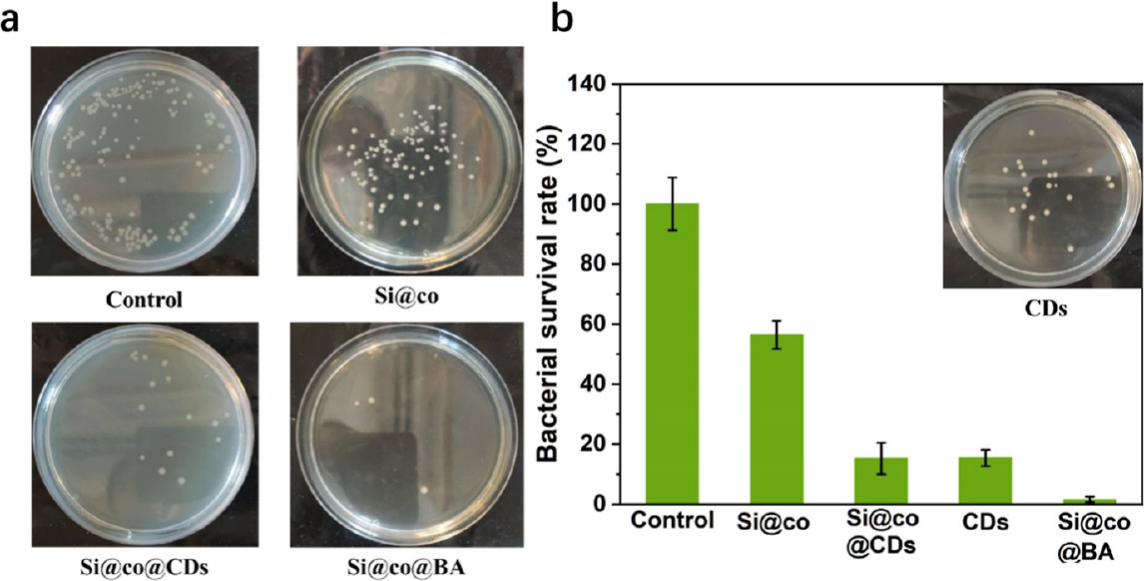
(a) Photographs and (b) corresponding cell survival rate of *E. coli* treated with 1 mg/mL of Si@co, Si@co@CDs, Si@co@BA, and 200 μg/mL of CDs.

QACs can interact with negatively charged cell membranes through electrostatic interactions, leading to strong cellular lysis, while copper-doped carbon dots can kill bacteria by destroying cell membranes and binding to bacterial proteins.^50^Figure 6 shows the antibacterial results of different nanocomposites to demonstrate the synergistic antibacterial effect. Compared with the control group, the bactericidal rate of Si@co against *E. coli* was 56.4% at a concentration of 1 mg/mL, mainly due to the positive charge of the cationic copolymer. The antibacterial abilities of CDs were also explored. As shown in Figure 6a, the CDs (200 μg/mL) exhibited a high antibacterial effect with a 15.4% bacterial survival rate. The strong antibacterial behavior of CDs may be attributed to their small size, enabling easy access to the cell membranes, and CDs could achieve 100% antibacterial efficiency at the same concentration (Figure S6a). When the CDs were loaded onto Si@co, their antibacterial ability is limited but can still kill bacteria by direct contact based on charge-induced physical destruction and reactive oxygen species-triggered oxidative stress.^41^ As a result, the bacterial survival rate with 1 mg/mL of the nanocomposite Si@co@CDs was found to be 15.2%, similar to that achieved with 200 μg/mL of CDs. This result indicates that only a small number of CDs need to be loaded onto the nanocomposites to achieve a bactericidal effect. For the nanocomposite containing both CDs and boronic acids (Si@co@BA), the bactericidal rate reached 99% with 1 mg/mL of the antibacterial materials. The mechanism of bacteria-killing using the Si@co@BA nanocomposite was further investigated by measuring reactive oxygen species during the bactericidal process. As shown in Figure S6b, *E. coli* showed bright green fluorescence after treatment with Si@co@BA, indicating that the CDs on the nanocomposite had generated some reactive oxygen species for bacteria-killing. These results suggest that both the CDs and the boronic acids contributed to enhancing the antibacterial efficiency of the cationic copolymer in a synergistic way. In addition, Live/Dead staining was carried out to further observe the status of *E. coli* after treatment (Figure S7a). For the bacteria treated with Si@co, half of the cells were dead, as indicated by the red fluorescence. Treatment with Si@co@CDs and Si@co@BA led to more obvious *E. coli* cell death. Additionally, the morphologies of *E. coli* after treatment were further observed by SEM (Figure S7b). The control group showed intact bacterial structures with smooth cell membrane surface, while partly wrinkled deformations appeared on the cell walls after Si@co treatment. In contrast, the bacteria treated with Si@co@CDs and Si@co@BA exhibited collapsed membranes and an abnormal cellular morphology, suggesting that the integrity of the bacteria was severely damaged.

### 3.3 Bacterial Binding Behavior of Si@co@BA

**Figure 7:**
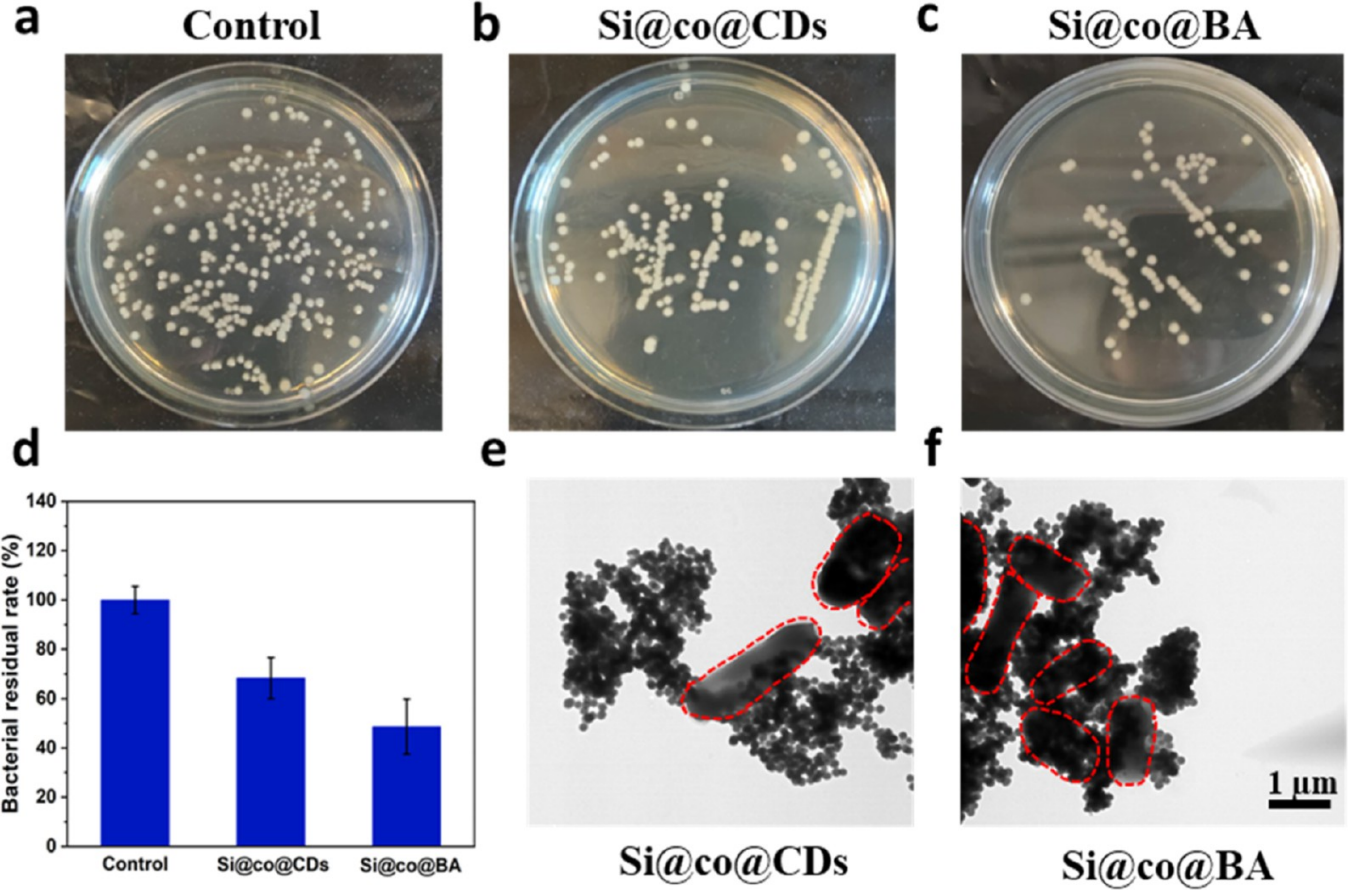
(a–c) Photographs and (d) corresponding residual rate of remaining *E. coli* in the medium after treatment with Si@co@CDs and Si@co@BA. TEM images of *E. coli* mixed with (e) Si@co@CDs and (f) Si@co@BA. Scale bar: 1 μm.

Boronic acids are known to bind to bacterial membranes by forming boronate ester bonds with the *cis*-diol structures on the extracellular polysaccharides on bacterial surface.^51^ In this work, Si@co@BA was designed to capture *E. coli* through formation of a boronic acid–diol complex and electrostatic interaction. To investigate the bacterial affinity of Si@co@BA for binding and labeling bacteria, a bacterial separation experiment was conducted. Si@co@BA was mixed with *E. coli* in PBS. After separation of the settlement of bacteria-Si@co@BA aggregates, the residual bacteria were quantified by plate counting. As shown in Figure 7a–d, both Si@co@CDs and Si@co@BA displayed significant bacterial trapping capacity, while Si@co@BA exhibited a higher bacterial trapping capacity than Si@co@CDs. This result can be attributed to the additional removal capability contributed by the boronic acid in Si@co@BA, in addition to the electrostatic interaction originated from the cationic copolymer.

The interaction between the nanocomposites and *E. coli* was further investigated by studying bacteria-nanocomposite aggregates using TEM. As shown in Figure 7e and f, the bacterial cells (red circles) treated with Si@co@CDs were found to be separated from the nanocomposites and formed several aggregates. For bacteria treated with Si@co@BA that contains boronic acid, the bacilliform *E. coli* was closely surrounded by many nanocomposites as the boronic acid molecules readily facilitated the formation of the nanocomposite-cell complex. Furthermore, the bacteria remained round shaped, and no apparent cellular damage was observed, indicating that the reduction of bacteria in the supernatant mainly resulted from molecular binding rather than bacteria killing.

### 3.4 Labeling and Imaging of Bacteria with Si@co@BA

**Figure 8:**
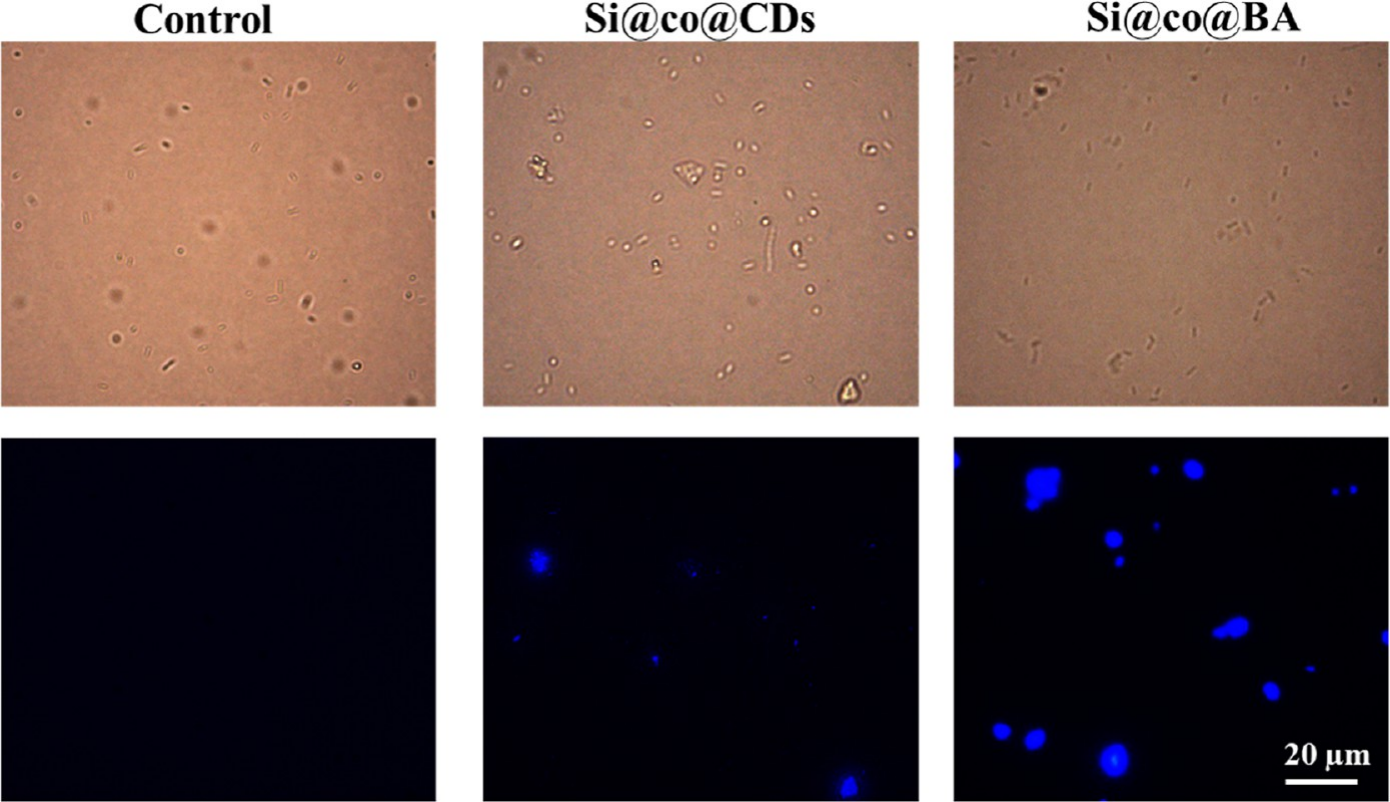
Fluorescent microscope images of *E. coli* treated with Si@co@CDs and Si@co@BA (scale bar: 20 μm; excitation: 405 nm).

The microbial binding and imaging capability of the fluorescent nanocomposites were assessed using fluorescence microscopy of *E. coli* treated with the nanocomposites. As shown in Figure 8, in the control group, the *E. coli* cells were invisible under fluorescence microscopy. After adding the two types of nanocomposites, the *E. coli* cells were effectively labeled with the nanocomposites and emitted intense blue fluorescence from the CDs. Flow cytometry was also conducted to evaluate the nanocomposites for bacterial fluorescent labeling. Bacterial cells typically exhibit low intensity autofluorescence due to the presence of endogenous fluorophores such as collagens, porphyrins, and flavins.^52^ As shown in Figure S8, *E. coli* cells in the control group emitted a weak background fluorescence. After the addition of Si@co@CDs and Si@co@BA nanocomposites, the bacterial cells showed a significantly higher fluorescence. The population-intensity peaks in the flow cytometry chart of the nanocomposite-treated *E. coli* right-shifted to form a broader peak compared to the control sample. The result indicates a strong attachment of the nanocomposites on the surface of the bacteria membrane even under the flow condition. As a result, the fluorescent nanocomposites may be used as potential fluorescence markers to detect living bacteria by flow cytometry based on their fluorescent imaging and bacterial binding capacity.

## 4 Conclusions

In conclusion, we have developed innovative multifunctional nanocomposites capable of bacterial binding, fluorescent imaging, and synergistic antibacterial activity by combining cationic copolymer brushes grafted onto silica nanoparticles with copper-doped CDs and boronic acid. These hybrid nanomaterials exhibit bacterial fluorescent imaging capabilities thanks to the incorporated CDs, enhanced affinity for binding bacterial surfaces through boronic acid binding and electrostatic interaction, and a synergistic antibacterial effect based on the positive charge of QACs and copper-ion-induced bactericidal action. Although the antibacterial ability of CDs is somewhat reduced after incorporation into the polymer brushes, the nanocomposite can still kill bacteria by direct contact based on charge-induced physical destruction and reactive oxygen species-triggered oxidative stress. The multifunctional nanocomposites open new possibilities for affinity separation, detection, and inhibition of pathogenic bacterial cells, shedding new light on the development of innovative antibacterial materials and other biological research platforms such as glycan labeling and synergistic treatment of cancer cells.

## Supporting Information Available

Details and additional results of binding analysis, boronic acid determination, bacteria cultivation, antibacterial activity assay, and characterization of Si@co@BA (PDF)
