# Supplementary material for: Cationic Polymer Brushes Functionalized with Carbon Dots and Boronic Acids for Bacterial Detection and Inactivation
Source: ACS Omega. 2025 Apr 2;10(14):14536–46. doi: 10.1021/acsomega.5c01507 (PMC12004185; doi:10.1021/acsomega.5c01507)
Supplement: Supplementary file 1 — ao5c01507_si_001.pdf [file ao5c01507_si_001.pdf]

Supporting Information

Cationic Polymer Brushes Functionalized with Carbon Dots and Boronic Acids for Bacterial Detection and Inactivation

Qicheng Zhang,[1] Si Chen,[2] Xiaoting Xue,[2] Solmaz Hajizadeh,[1] Tomohiko Yamazaki,[3] Lei Ye[1,*]

[1] Division of Pure and Applied Biochemistry, Department of Chemistry, Lund University, 22100 Lund, Sweden

[2] Polymer & Materials Chemistry, Department of Chemistry, Lund University, 221 00 Lund, Sweden

[3] Research Center for Macromolecules and Biomaterials, National Institute for Materials Science (NIMS), 305-0047 Tsukuba, Japan

Corresponding author:

Lei Ye, Email: lei.ye@tbiokem.lth.se

Cultivation of bacteria

Gram-negative *Escherichia coli* (*E. coli*, TG1) were cultured in 1.5 mL tubes with 1 mL LB medium at 37 °C overnight. The cells were washed with PBS solution for 3 times by centrifugation at 12000 rpm for 1 min, and finally resuspended in 1 mL of PBS. The counting of bacteria is based on a positive correlation between the concentration and the optical density of bacteria at 600 nm (OD_{600}). For binding experiments, the OD_{600} value of bacteria samples was diluted to 0.1 with PBS.

Detection of boronic acid groups on polymers using ARS

Si@co@BA (5 mg) was suspended in 2 mL of ARS solution (0.1 mM) prepared in phosphate buffer (20 mM, pH 7.4) and sonicated for 10 minutes. The fluorescence spectrum of the mixture was detected with an excitation wavelength at 470 nm. For comparison, Si@co@BA dispersion and ARS solution were used as controls and measured with the same procedure.

Antibacterial activity assay

Different concentrations of Si@co@BA dispersion or PBS were mixed with *E. coli* suspension to prepare the final concentrations of Si@co@BA at 0.25, 0.5, 1 and 2 mg/mL and an OD_{600} value of *E. coli* at 0.1. The mixture was gently rotated (30 rpm) at room temperature for 3 h. After all the suspensions were diluted with PBS by 10^5 times, 50 μL of the dilution was transferred to LB agar plate for spreading and incubated at 37 °C overnight before collecting images of the agar plates. In order to compare the antibacterial effects of different materials, 0.5 mL of Si@co, Si@co@CDs, Si@co@BA (2 mg/mL) and CDs (0.2, 2 mg/mL) were added into 0.5 mL of *E. coli* suspension (OD_{600} = 0.2). Afterward, the incubation and plate counting assay were performed following the procedures as described above.

The bacterial cells after various treatments were further collected by centrifugation at 10000 rpm for the bacterial live/dead assays. Bacteria were co-stained with SYTO 9 and PI fluorescent dyes for 30 min under dark conditions. After staining, the bacteria

dispersions were washed with PBS for three times to remove excess dye and imaged under fluorescence microscope. Additionally, the bacterial dispersions after separation were resuspended in PBS and mixed with 2.5 vol% glutaraldehyde for 12 h. Then, bacteria were dropped on a clean glass slide followed by air drying overnight and treated by serial dehydration with increasing concentrations of ethanol (25%, 50%, 75%, 80%, 90% and 100%) for 30 min. The morphologies of bacteria were observed by SEM after sputter-coating the cells with gold.

Bacteria binding analysis

Si@co@CDs and Si@co@BA (2 mg) were dispersed in 2 mL of *E. coli* suspension (OD_{600} = 0.3) in PBS. The samples were gently rotated (30 rpm) at room temperature for 30 min and 1 mL of the mixture was added into 1 mL of glutaraldehyde solution (5% v/v in PBS). After incubation at 20 °C for 1 h, the bacteria were isolated by centrifugation and washed thoroughly with PBS buffer several times to remove glutaraldehyde. The bacteria were resuspended in 1 mL of PBS and observed by TEM; The remaining half of the suspension was then centrifuged at 2000 rpm (270 g) for 6 s, followed by natural settlement for 30 min. Afterward, the supernatant was diluted and spread on the agar plate, from which the amount of the unbound bacteria was counted.

Fluorescence microscopy

E. coli cell suspension (OD_{600} = 0.3 in 1 mL of PBS) was mixed with Si@co@CDs and Si@co@BA dispersion (200 μg/mL in 1 mL of PBS buffer) and rotated (30 rpm) at room temperature for 10 min. After the centrifugation, the bacteria were washed with PBS for several times and then gently dropped on a glass slide. The fluorescence of the samples was observed by a Nikon Eclipse Ci fluorescence microscope.

Flow cytometry

E. coli cell suspension (OD_{600} = 0.1 in 0.9% NaCl) was mixed with Si@co@CDs and Si@co@BA (1 mg/mL) and rotated (30 rpm) at room temperature for 10 min. The fluorescence intensity of the bacterial cells was detected by a MACSQuant VYB Flow

Cytometer (Miltenyi Biotec, GmbH) using a violet laser with excitation at 405 nm and detection through a 450/50 nm (V1 channel) filter. Quality control was performed prior to each experiment with MACSQuant Calibration Beads (Miltenyi Biotec, GmbH). Flow cytometry raw data was exported from the MACSQuantify software and analyzed by FlowJo software (v10; Treestar, Inc., San Carlos, CA).

ROS detection

E. coli cell suspension (OD_{600} = 0.2) was mixed with Si@co@BA (2 mg/mL) in a ratio of 1:1 and the mixture were shaken at 37 °C for 3 h. Afterwards, *E. coli* was stained with DCFH-DA for 30 min under dark conditions. After staining, the bacteria dispersions were imaged under fluorescence microscope.

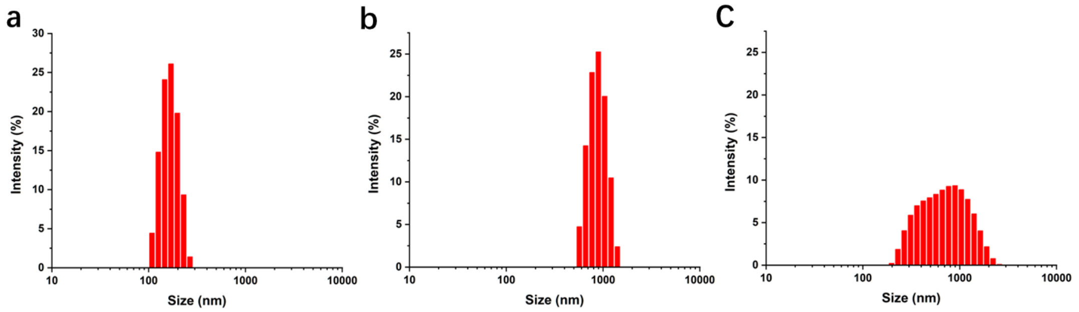

Figure S1. DLS results of (a) Si@BiBB, (b) Si@co, and (c) Si@co@BA.

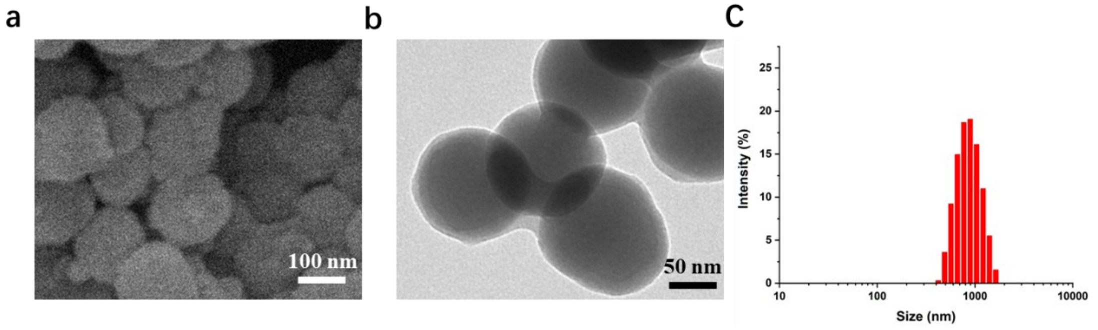

Figure S2. (a) SEM, (b) TEM and (c) DLS results of Si@co@CDs.

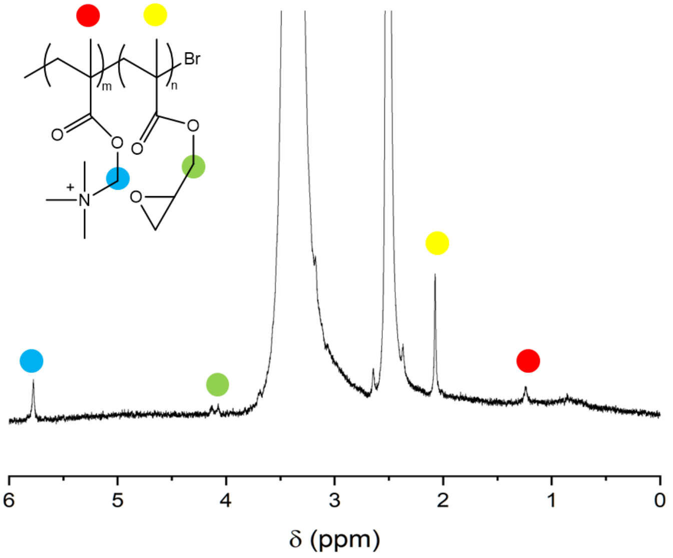

Figure S3. ^{1}H NMR spectrum of Si@co.

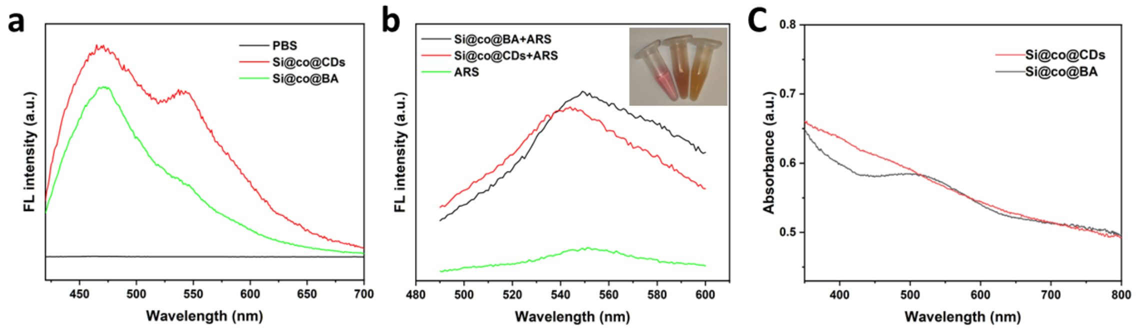

Figure S4. (a) Fluorescence spectra of PBS, Si@co@CDs and Si@co@BA (Excitation: 400 nm). (b) Fluorescence spectra and (c) UV-vis spectra of Si@co@CDs and Si@co@BA suspensions mixed with ARS (Excitation: 470 nm).

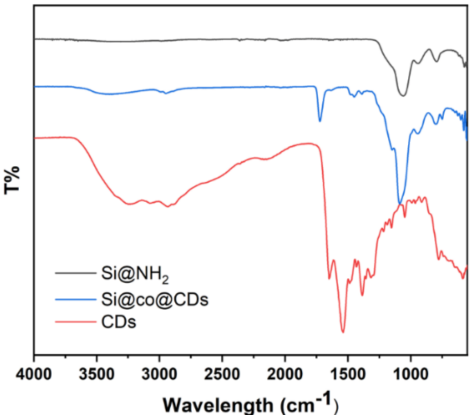

Figure S5. FTIR spectra of Si@NH_2, Si@co@CDs and CDs.

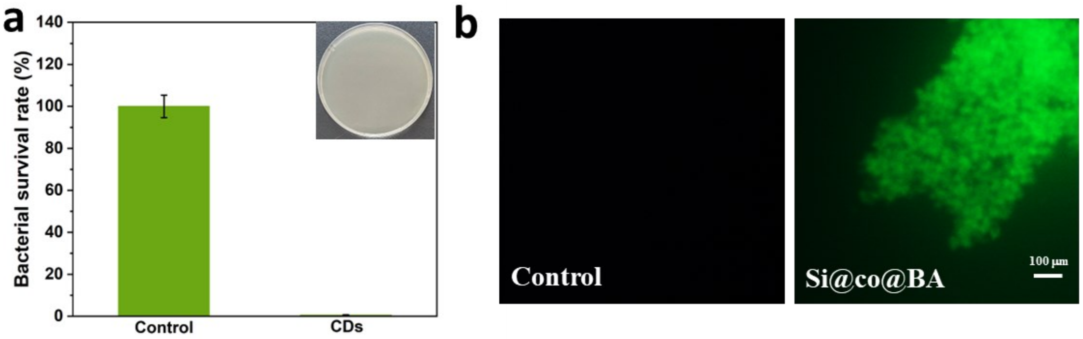

Figure S6. (a) Photographs and corresponding cell survival rate of *E. coli* treated with 1 mg/mL of CDs. (b) ROS detection of *E. coli* stained with DCFH-DA after treated with Si@co@BA (Scale bar: 100 μm).

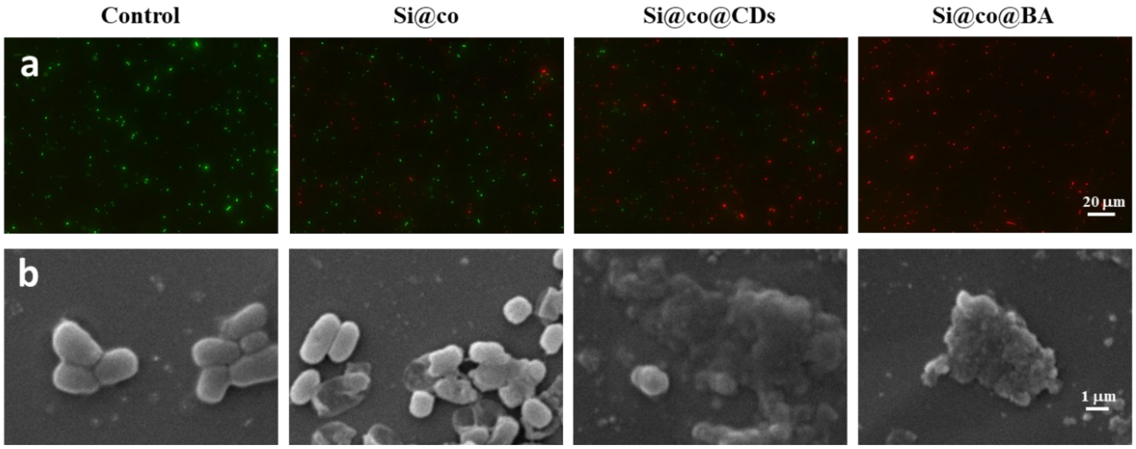

Figure S7. (a) Live/dead bacterial staining fluorescent images and (b) SEM images of *E. coli* after incubation with Si@co, Si@co@CDs and Si@co@BA (Scale bar in a: 20 μm and b: 1 μm; Excitation for SYTO 9/PI: 488/561 nm).

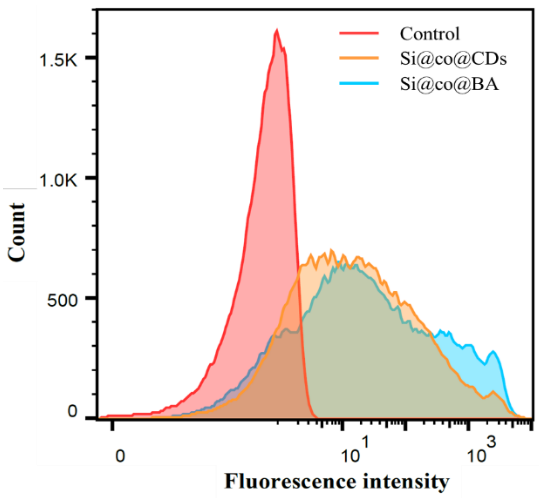

Figure S8. Flow cytometry analysis of *E. coli* incubated with Si@co@CDs and Si@co@BA (Excitation: 405 nm).
